# Four-Year Experience With the ALTO Stent Graft in Abdominal Aortic Aneurysms With Hostile Features

**DOI:** 10.7759/cureus.95323

**Published:** 2025-10-24

**Authors:** Yasser Elsayed, Mahmoud Bakheet, Namareq Ahmad, Thamer Babiker, Bilal Rawshdeh, Mohamed Banihani

**Affiliations:** 1 Vascular Surgery, Lancashire Teaching Hospitals NHS Foundation Trust, Preston, GBR

**Keywords:** abdominal aortic aneurysms (aaa), alto stent graft, aneurysm sac expansion, endovascular aortic repair (evar), hostile neck aaa, sac stabilization

## Abstract

Introduction: Low-profile stent grafts with polymer ring sealing technology expanded the use of endovascular aortic repair (EVAR) to include short conical necks and small access vessels in general. We examined the use of the ALTO stent graft (Endologix LLC, Irvine, CA) in our regional vascular centre to assess its real-life performance in cases deemed not suitable for standard stenting. This was an independent, single-centre study with no financial or material involvement from the device manufacturer.

Methods: This was a retrospective observational review of all consecutive patients treated with the ALTO stent graft between May 2020 and June 2024. Patient demographics, anatomical and radiological features, re-interventions, and general follow-up data were collected. Primary endpoints included long-term survival and freedom from Type Ia endoleak. Secondary outcomes included sac expansion and reinterventions.

Results: Forty-nine patients were included, of whom 71.4% were not fit for open repair. Median follow-up was 48 months. Half of the patients had a flared/conical neck, 20% had significant neck thrombus, and 30% had a small aortic bifurcation or small iliac vessels. The median length of the conical/flared neck was 10.5 mm (4-48 mm). Median maximal aortic neck diameter was 25.5 mm (19.5-31 mm). Median iliac artery diameter was 6.5 mm (4-9 mm). The technical success rate was 98%, with only one patient converted to open. One patient died within 30 days after discharge from chronic obstructive pulmonary disease (COPD)/COVID. Freedom from reintervention at 12, 24, 36, and 48 months was 100%, 100%, 100%, and 85.71%, respectively. One patient developed a Type III endoleak after four years. We observed no aneurysm-related mortality.

Conclusion: The ALTO stent-graft system demonstrates an effective and durable seal in abdominal aortic aneurysm (AAA) cases with hostile features, with outcomes comparable to standard EVAR, expanding the options for patients less fit for open repair.

## Introduction

Abdominal aortic aneurysms (AAA) represent a significant cause of morbidity and mortality, particularly in elderly populations. The evolution of endovascular aortic aneurysm repair (EVAR) offered a minimally invasive alternative to open surgical repair, leading to decreased perioperative morbidity, shorter hospital stays, and faster recovery. Since its introduction in the 1990s, EVAR has become the first-line treatment for anatomically suitable aneurysms, especially in older and high-risk surgical patients [1].

However, the applicability of EVAR is limited by anatomical features, particularly the characteristics of the proximal aortic neck. Hostile neck features, including short necks (<10-15 mm), angulation >60 degrees, conical or flared neck configurations, thrombus load, and severe calcification, are associated with higher rates of technical failure, endoleaks, and the need for secondary interventions. In addition, small or diseased access vessels and narrow aortic bifurcations add more challenges to the correct deployment of conventional EVAR devices [2].

To try to solve these limitations, newer-generation endografts such as the ALTO abdominal stent graft system (Endologix LLC, Irvine, CA, USA) have been developed. ALTO incorporates a new sealing technology using a polymer-filled ring located just below the renal arteries, allowing for more flexible treatment of challenging neck anatomies. Additionally, its low-profile delivery system enables good access through small or tortuous iliac arteries, thereby expanding EVAR eligibility to a large number of patients [3].

This study evaluates the real-world experience with the ALTO stent graft system in a single tertiary vascular centre. We present a comprehensive analysis of procedural feasibility, clinical effectiveness, and long-term outcomes over a four-year period, with particular focus on patients with hostile anatomical features previously considered outside the instructions for use (IFU) of standard EVAR [4].

## Materials and methods

Study design and patient selection

This study was designed as a retrospective observational review conducted at Lancashire Teaching Hospitals NHS Foundation Trust, a single tertiary vascular centre in Preston, UK. All consecutive patients who underwent EVAR using the ALTO stent graft system between May 2020 and June 2024 were included. The aim was to assess the real-world performance of the device in patients with challenging aortic anatomies, including those outside the IFU of conventional EVAR systems.

Patient selection for the ALTO device was based on a detailed computed tomography angiography (CTA) assessment and discussion in a multidisciplinary team (MDT) meeting. The device was offered to patients who were anatomically unsuitable for standard EVAR due to hostile proximal neck or access vessel features or who were considered unfit for open surgical repair because of significant comorbidities or frailty.

Patients were included if they had infrarenal AAAs with hostile anatomical features, such as a short proximal neck less than 15 mm, severe neck angulation greater than 60 degrees, conical or flared neck configuration, or the presence of significant mural thrombus or circumferential calcification at the neck. In addition, patients with a narrow aortic bifurcation, small iliac access vessels, or tortuous iliac anatomy were also considered eligible for the use of the ALTO device.

Patients were excluded if they had suprarenal, thoracoabdominal, mycotic, or inflammatory aneurysms or if they presented with ruptured or symptomatic leaking aneurysms. Those with anatomical characteristics that precluded safe deployment of the ALTO device, even within its specific IFU, were also excluded from this analysis.

All procedures were discussed and approved in the vascular MDT meeting prior to intervention. Final device selection and operative planning were made according to anatomical suitability and consultant discretion.

Data collection and variables

All pertinent data were meticulously abstracted from a comprehensive review of the institution's electronic medical records, detailed operative reports, and digital picture archiving and communication system (PACS) imaging archives. The collected dataset encompassed baseline demographic information, including patient age, sex, and relevant medical comorbidities. Aneurysm morphology was thoroughly characterised by recording the maximal aortic diameter, proximal neck length and angulation, the burden of intraluminal thrombus and calcification, as well as the presence of concomitant iliac artery aneurysms and access vessel challenges. Procedural parameters documented included the achievement of technical success and any intraoperative complications. Follow-up data focused on the incidence of endoleaks, the necessity for reinterventions, changes in aneurysm sac size, and all-cause and aneurysm-related mortality.

Definitions and outcome measures

For the purpose of this study, technical success was defined as the successful deployment of the ALTO stent graft at the intended implantation site with the absence of a Type I or Type III endoleak on the final completion angiogram obtained in the operating suite. Endoleaks were classified according to the criteria by White et al. as Type I (graft attachment site), Type II (branch vessel), Type III (graft defect), Type IV (graft porosity), and Type V (endotension) [5]. The primary endpoints of this analysis were designed to evaluate long-term device performance and patient safety, specifically focusing on four-year overall survival, freedom from aneurysm-related mortality, and freedom from Type Ia endoleak. Secondary endpoints of interest included the occurrence of Type Ib and Type III endoleaks, significant aneurysm sac expansion defined as an increase greater than five millimetres, and the rate of secondary reinterventions throughout the follow-up period. All grading systems and scales utilised are standard, validated tools in vascular surgery and were freely applied for this academic research.

Statistical analysis

Given the observational and descriptive nature of this study, data are primarily presented using descriptive statistics. Categorical variables are expressed as counts and percentages, while continuous variables are presented as means with standard deviations or medians with interquartile ranges, depending on the distribution of the data. For time-to-event outcomes, such as overall survival and freedom from reintervention, survival analyses were performed using the Kaplan-Meier method to generate estimates over the study period. Patients who underwent endovascular AAA repair using the ALTO stent graft were prospectively enrolled. Baseline demographic, clinical, and anatomical characteristics were recorded. Risk stratification was performed using the Glasgow Aneurysm Score (GAS) [6], a validated scale that predicts perioperative mortality and morbidity in AAA patients. The GAS was calculated according to the criteria described by Biancari et al. (2003) [7] and Baas et al. (2008) [8]. Other relevant scoring tools were referenced as needed, with their original publication cited at first use. All scoring systems employed in this study are free for academic use as per their primary literature and have been cited accordingly [7].

Ethical considerations

This study used anonymised retrospective data obtained from routine clinical records. No patient identifiers were collected, and no direct patient involvement occurred. In line with institutional policy, formal ethical approval was not required.

## Results

A total of 49 patients underwent EVAR using the ALTO stent graft. The mean GAS in this cohort was 76.2 years (range 58-87 years) with a 5:1 male:female ratio.

Anatomical features

**Figure 1:**
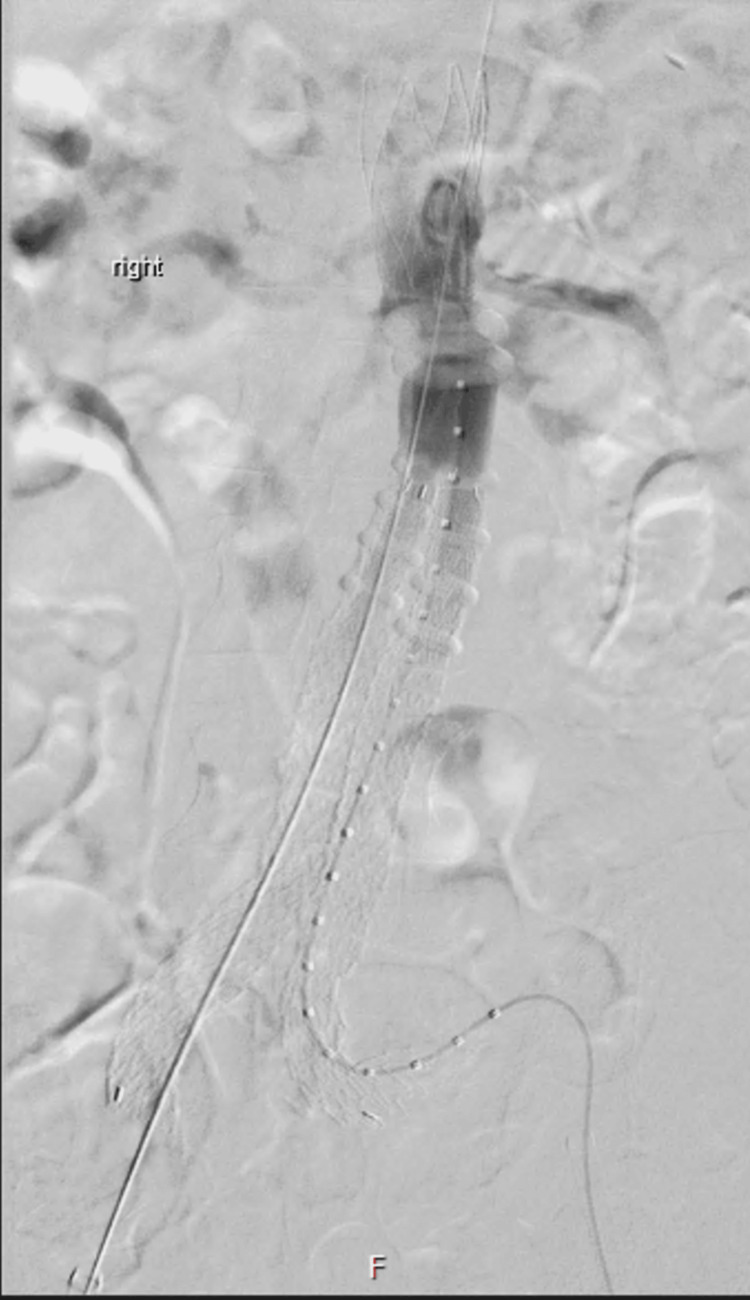
Angiogram after deployment of the ALTO device

**Figure 2:**
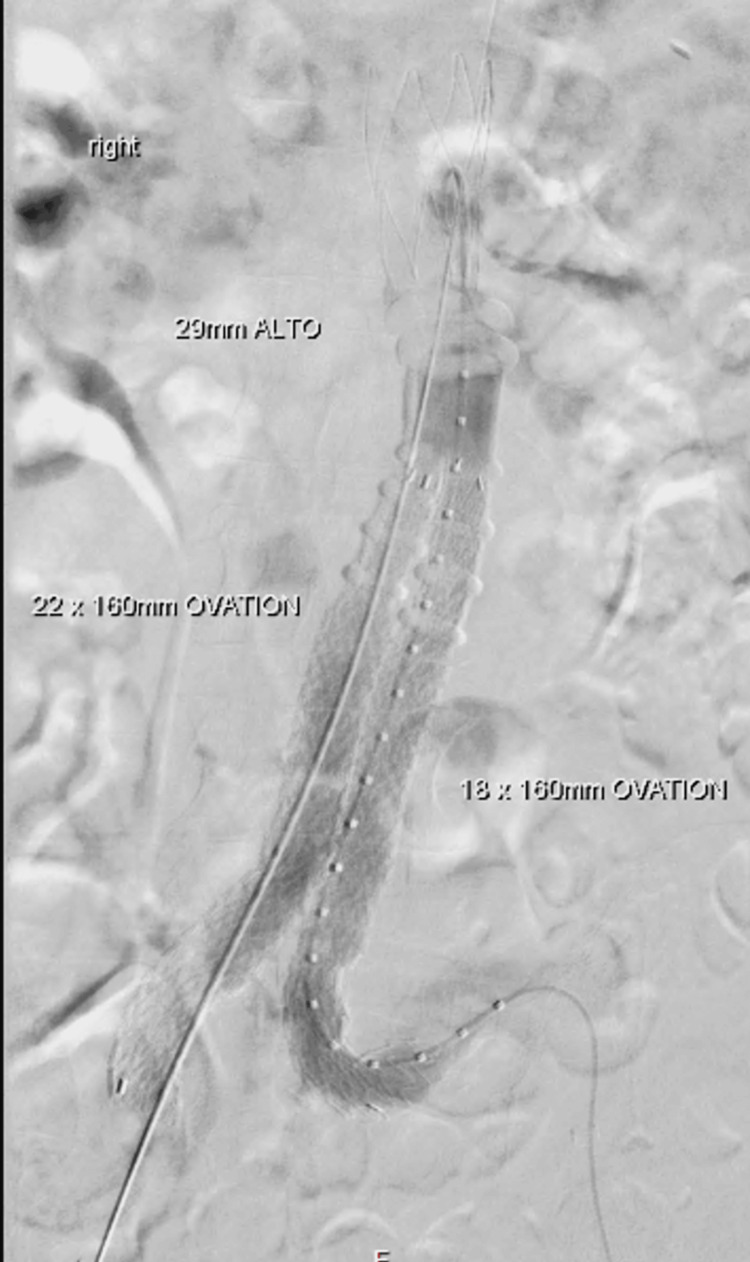
Angiogram after deployment of the ALTO device

The median maximal aneurysm diameter was 60 mm (49-100 mm). Twenty-five patients (51%) had a flared/conical neck. The median length of the conical/flared neck was 10.5 mm (4-48 mm), and the median maximal aortic neck diameter was 25.5 mm (19.5-31 mm). One third of the patients had a small aortic bifurcation/iliac vessel. The median of small iliac diameter was 6.5 mm (4-9 mm). Figures 1, 2 showed an angiogram after deployment of the ALTO device.

Operative and early outcomes

**Figure 3:**
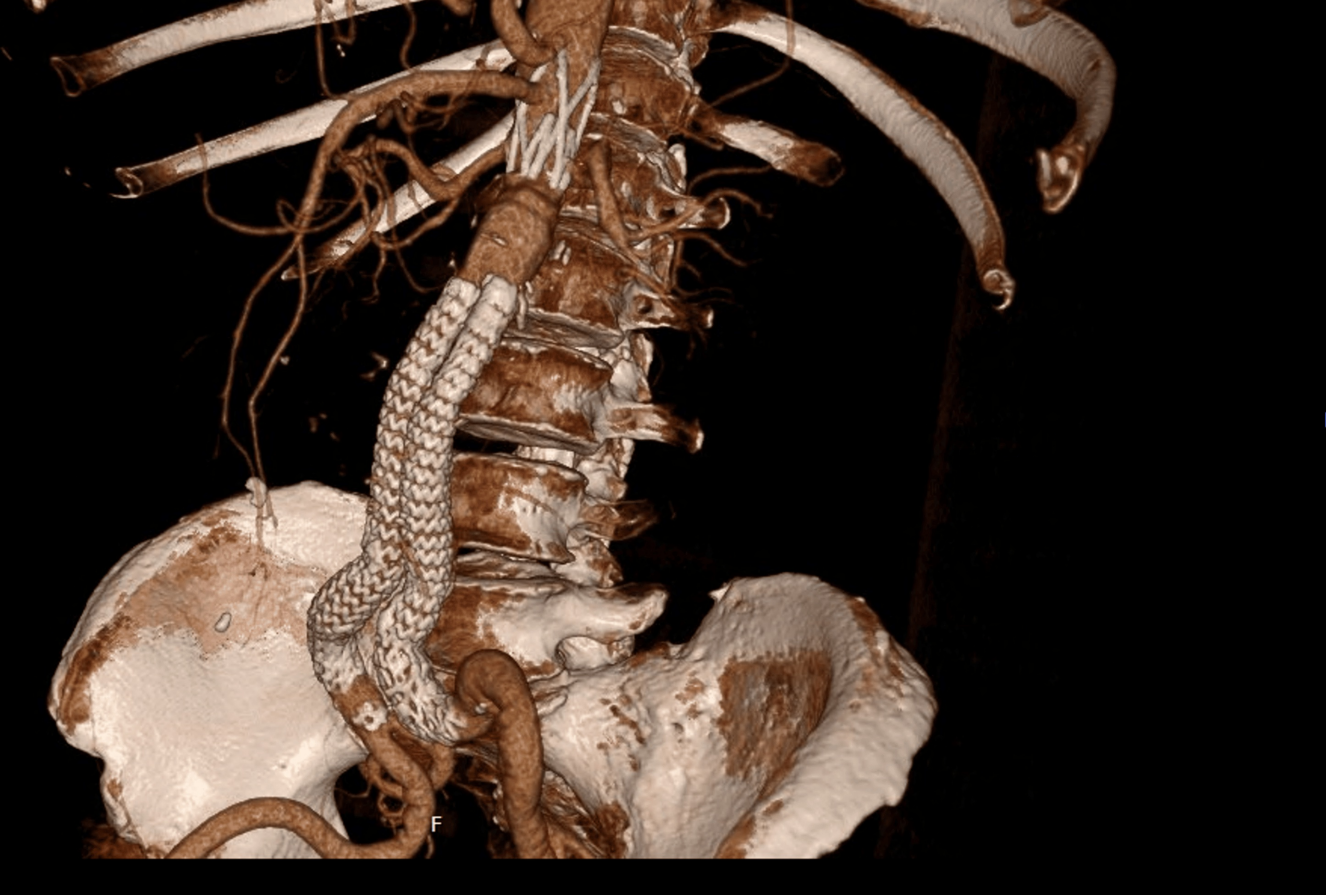
3D view of the ALTO stent 30 days postoperatively

The ALTO device was successfully deployed in 48 of 49 cases (98%). We encountered only one intraoperative Type III endoleak that was managed by open conversion, classified according to White et al. [5]. There was only one case of 30-day mortality in a patient who succumbed to chronic obstructive pulmonary disease (COPD)/COVID at home one week after discharge. Figure 3 shows a 3D view of the ALTO stent 30 days postoperatively.

Follow-up and four-year outcomes

No patient developed Type Ia endoleaks or sac expansion >5 mm during the follow-up period. We observed no aneurysm-related mortality, but overall mortality was 14%. One patient died at 30 days (COVID-related), four within two years, one during year three, and one during year four. 

**Figure 4:**
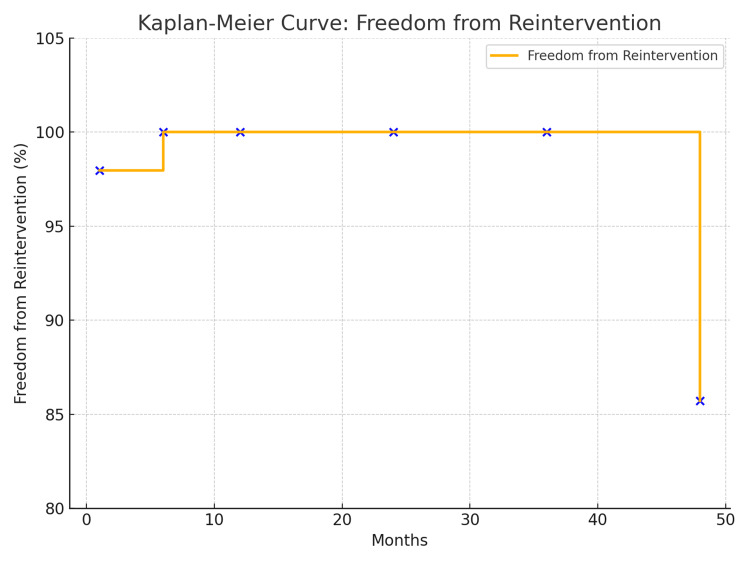
Kaplan-Meier Curve for freedom from reintervention

As demonstrated in Figure 4, freedom from reintervention at one, six, 12, 24, 36, and 48 months was 97.95%, 100%, 100%, 100%, 100%, and 85.71%, respectively. Only one patient developed a Type III endoleak at 48 months.

## Discussion

The ELEVATE IDE trial demonstrated that ALTO achieved 99% technical success and 97.4% freedom from Type Ia endoleak at 12 months. Our series parallels these outcomes and extends them to four years, demonstrating long-term durability. While ELEVATE IDE had strict inclusion criteria and controlled environments, our real-world cohort included patients with more severe anatomical challenges and comorbidities, underscoring the robustness of ALTO in everyday practice [9]. 

In our cohort, the ALTO stent graft system has provided a safe and durable treatment for infrarenal AAAs in patients with challenging anatomical criteria, with a technical success rate of 98% and no Type Ia endoleaks over four years. Our findings support the use of ALTO in challenging aortic anatomies outside IFU for conventional EVAR stents. These results are consistent with previous studies showing that the ALTO device, with its new polymer-sealing ring technology, provides an improved seal zone and greater anatomic flexibility, particularly in short or conical/flared aortic necks [10-11]. 

In our cohort, around two-thirds of the patients had one or more anatomical challenges, such as flared necks, thrombus burden, or small access vessels. Traditional endografts typically require a neck length of at least 15 mm for optimal sealing, while the ALTO system permits sealing in necks as short as 7 mm, provided the infrarenal angulation and thrombus burden are not prohibitive [12]. This broader anatomical applicability directly translates into increased eligibility for endovascular treatment in a population often considered unfit for open repair. 

Our results compare favourably with recent multicentre registry data. Pitros et al. reported a technical success of 99% and freedom from Type Ia endoleak at 12 months in 97.4% of cases [11]. These findings were replicated in our cohort with an extended reintervention-free success rate of 85.7% at 48 months, confirming the real-world durability of the device, particularly in anatomically complex patients. 

One of the distinguishing features of ALTO is its deployment accuracy and post-deployment stability, which is crucial in short or angulated necks. The polymer-sealing ring is actively deployed just below the renal arteries and moulded to the shape of the aortic neck at that level, allowing not only for precise positioning but also a tension-free sealing mechanism that avoids the ongoing radial force expansion related to the traditional self-expanding devices. In our series, this mechanism likely contributed to the absence of late Type Ia endoleaks, which remains a significant concern with standard stent grafts in hostile necks. Gradual neck dilatation is a well-recognised cause of long-term failure in standard EVAR that has been shown to be less pronounced in the ALTO device, as recently demonstrated by Ichihashi et al. [10]. 

The only case of endoleak observed in our study was a delayed Type III endoleak at four years. This was treated successfully with relining the right iliac stent. Type III endoleaks, typically resulting from component separation or fabric tears, are rare but emphasise the need for ongoing surveillance even in technically successful EVAR cases. Our data also suggest that sac stability was achieved in all surviving patients, with no aneurysm growth >5 mm observed, a crucial metric correlating with successful aneurysm exclusion and long-term protection against rupture. 

Notably, the majority of patients treated with the ALTO device in our cohort were deemed unfit for open surgery. Over 70% of the patients had significant comorbidities such as COPD, frailty, or cardiovascular disease that precluded open repair. In this high-risk group, the ALTO device enabled the provision of life-saving therapy where no other viable option existed. A recent health economics analysis by Ouriel et al. (2023) further supports the use of polymer-sealing stent grafts in such populations, demonstrating favourable cost-effectiveness and reduced reintervention costs over a five-year horizon [11]. 

The ALTO’s design mitigates many of the “hostile neck” challenges, but it requires careful patient selection and precise intraoperative technique. The learning curve, although manageable, emphasises the need for adequate training in sizing and polymer fill technique to avoid maldeployment or endoleak [13]. Comparative device studies have also shown a favourable profile for low-profile systems when compared to standard EVAR [14]. 

We acknowledge the limitations of our study, as it is retrospective, single-centre, and observational in design, limiting the ability to draw definitive causal inferences. We also recognise the absence of a comparator group receiving alternative stent grafts, but the unique features of the ALTO device make it almost impossible to compare, especially in flared/conical necks, where no other stent graft can be used. Additionally, follow-up imaging was not standardised across all patients due to logistical constraints during the COVID-19 pandemic [15]. 

Nonetheless, the real-world nature of our cohort adds clinical value. Our patients reflect typical scenarios in vascular practice - those with frailty, challenging anatomy, and limited access options, often excluded from randomised trials. By demonstrating that ALTO can be safely used with excellent midterm outcomes in this population, our findings support broader adoption of the device [16]. 

Future research should focus on longer-term follow-up (beyond five years), registry collaboration across centres, and randomised comparisons between ALTO and other new-generation EVAR systems. Additionally, the incorporation of advanced imaging techniques such as intraoperative fusion imaging and postoperative 4D flow MRI may further optimise outcomes and enhance post-EVAR surveillance strategies [17]. 

Clinical implications

ALTO enables safe EVAR stenting in traditionally “hostile” anatomy and is also suitable for elderly and comorbid patients, reducing operative risk. It may lower long-term healthcare costs by reducing secondary interventions. 

Limitations

This study is limited by its retrospective and single-centre design, which restricts the ability to establish causation or broad generalisability. The sample size is relatively small, reflecting the real number of ALTO cases performed at our regional vascular centre during the study period. Follow-up duration varied slightly between patients, as this depended on routine clinical scheduling. Nevertheless, the results provide a genuine reflection of real-world outcomes in a medium-volume vascular unit. Future collaboration with high-volume centres and larger multicentre studies will be valuable to confirm these results and refine patient selection criteria.

Future directions

Larger, multicentre prospective studies with extended follow-up will be essential to confirm our findings and further define the role of the ALTO stent graft in vascular practice. 

## Conclusions

The ALTO stent graft provides a safe and durable EVAR option for patients with hostile aortic anatomy. Over four years, we observed high technical success, low reintervention rates, and no aneurysm-related mortality, supporting its use in anatomically complex cases and in patients unfit for open repair. Future collaboration with high-volume centres and larger multicentre studies will be valuable to confirm these results and refine patient selection criteria.
